# Supplementary material for: Effects of a Short Daytime Nap on the Cognitive Performance: A Systematic Review and Meta-Analysis
Source: Int J Environ Res Public Health. 2021 Sep 28;18(19):10212. doi: 10.3390/ijerph181910212 (PMC8507757; doi:10.3390/ijerph181910212)

Supplementary Figures

Figure S1. Summary of meta-analyses on cognitive performance for each cognitive function at baseline (t0) between groups

Each summary of meta-analysis is presented in three conditions: global model with all the studies, after exclusion of outliers (studies not evenly distributed around the funnel plot) and with only randomized controlled trials

Each summary of several meta-analysis is represented in the forest-plot by a dot on a horizontal line. The black dots represent the pooled-effect estimate (pooled effect size - ES), and the length of each line around the dots represent their 95% confidence interval (95CI). Shorter lines represent a narrower 95CI thus higher precision around pooled-ES. Conversely, longer lines represent a wider 95CI and less precision around pooled-ES. An overall summary of the results of the meta-analyses pooled-estimate (result of the overall meta-analysis) is represented by a blue lozenge at the end of the graph. The black solid vertical line represents the null estimate (with a value of 0 for pooled-ES). Horizontal lines that cross the null vertical line represent the non-significant overall summary of the meta-analysis.

		n studies (subgroups)	I-squared (%)	Effect size (95% CI)	Weight (%)
**By type of performance**					
**Global model**	Memory	7 (45)	13.9	0.02 (-0.11 to 0.16)	52.9
	Alertness	5 (28)	30.9	-0.07 (-0.27 to 0.12)	32.8
	Executive functions	6 (10)	24.6	-0.13 (-0.41 to 0.14)	14.3
	**Overall**	11 (83)	21.2	**-0.03 (-0.14 to 0.07)**	100
**After exclusion of outliers**	Memory	7 (43)	0.0	-0.02 (-0.14 to 0.11)	53.5
	Alertness	4 (26)	0.0	0.01 (-0.15 to 0.18)	32.4
	Executive functions	5 (9)	0.0	-0.03 (-0.28 to 0.23)	14.0
	**Overall**	11 (78)	0.0	**-0.01 (-0.10 to 0.09)**	100
**only RCT**	Memory	5 (42)	13.3	0.04 (-0.11 to 0.18)	73.5
	Alertness	2 (8)	0.0	-0.13 (-0.37 to 0.10)	21.4
	Executive functions	2 (2)	13.8	-0.69 (-1.21 to -0.17)	5.1
	**Overall**	6 (52)	17.2	**-0.04 (-0.16 to 0.09)**	100

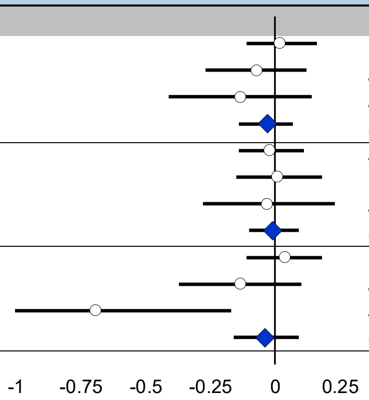

Figure S2. Funnel plot - Meta-analysis on overall performance between groups at baseline (t0), at t1 and on comparison between t1 and t0 for each group (nap and control) and stratified on type cognitive function

SMD: Standardized Mean Difference; se(SMD): standard error of standardized mean difference

Each dot represents a single study, with its corresponding effect size (x axis) and its associated standard error of the effect estimate (y-axis). Large high-powered studies are placed towards the top, and smaller low-powered studies towards the bottom. The plot should ideally resemble a pyramid or inverted funnel, with scatter due to sampling variation. Studies outside funnel plot are likely to present bias (Sterne JA, Sutton AJ, Ioannidis JP, Terrin N, Jones DR, Lau J, Carpenter J, Rücker G, Harbord RM, Schmid CH, Tetzlaff J, Deeks JJ, Peters J, Macaskill P, Schwarzer G, Duval S, Altman DG, Moher D, Higgins JP. Recommendations for examining and interpreting funnel plot asymmetry in meta-analyses of randomised controlled trials. BMJ. 2011 Jul 22;343:d4002. doi: 10.1136/bmj.d4002).

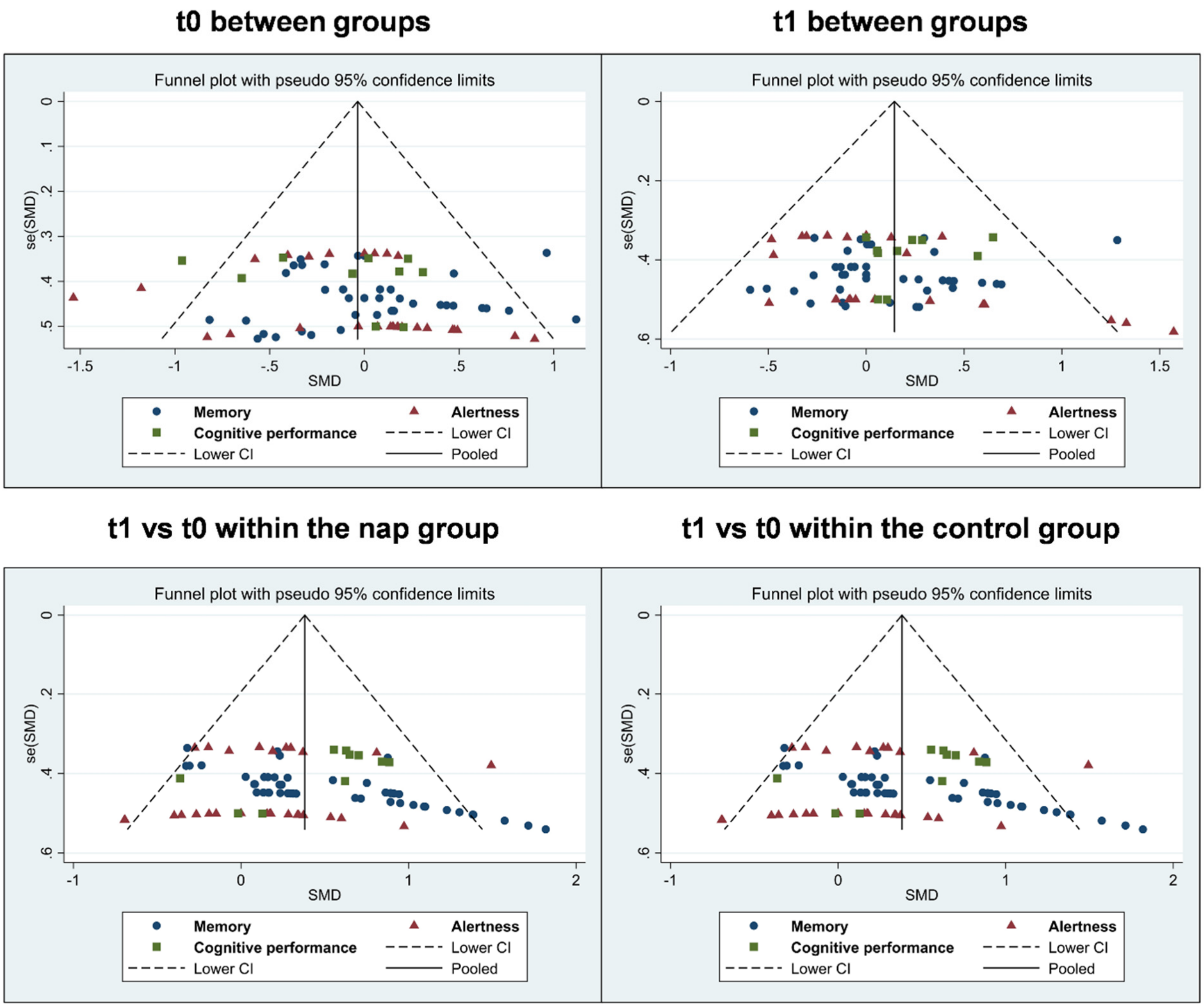

Figure S3. Meta-analysis on overall effects of nap within the nap group (t1 vs t0) stratified on time of analysis

95%CI: 95% confidence intervals; AOT, Arrow orientation task; CPT II, Conner's Continuous Performance Test Version 5; DSB, Digit span backwards task; EEG, Electroencephalographic; MAT, Motor adaptation task; MTT, Mirror Tracing Task; PAL, Paired associates learning; SUPA, Semantically unrelated paired associates; TAP, Test of Attentional Performance; VLMT, Verbal learning and memory test; WPT, Word-pair task. Each individual study or subgroup used in the meta-analysis is represented in the forest-plot by a dot on a horizontal line. The black dots represent the effect estimate (effect size - ES), and the length of each line around the dots represent its 95% confidence interval (95CI). Shorter lines represent a narrower 95CI thus higher precision around study ES, usually found in larger studies. Conversely, longer lines represent a wider 95CI and less precision around ES usually found in smaller studies. An overall summary pooled-estimate (result of the meta-analysis) is represented by a blue lozenge at the end of the graph. The black solid vertical line represents the null estimate (with a value of 0 for ES) and the red dotted vertical line represents result of the meta-analysis (pooled-estimate). Horizontal lines that cross the null vertical line represent non-significant studies.

Study	n	Age, years (mean±SD)	Sex (% male)	Nap opportunity (min)	Type of cognitive function	Type of test	Time between nap & test (min)	Effect size (95CI)	Weight %
**Less than 30 minutes**									
Amin 2012, cognitive task 1	29	29±1.9	61.0%	20	Alertness	CPT II	25	0.81 (0.13, 1.49)	1.42
Amin 2012, cognitive task 2	29	29±1.9	61.0%	20	Cognitive performance	CPT II	25	0.63 (-0.04, 1.30)	1.44
Amin 2012, cognitive task 3	29	29±1.9	61.0%	20	Cognitive performance	CPT II	25	0.55 (-0.11, 1.22)	1.45
Amin 2012, EEG recording	29	29±1.9	61.0%	20	Alertness	EEG recording	25	1.49 (0.75, 2.24)	1.29
Asaoka 2010, cognitive task 1	8	24±1.1	75.0%	60	Alertness	AOT - compatible stimuli	1,5	-0.27 (-1.25, 0.72)	0.89
Asaoka 2010, cognitive task 2	8	24±1.1	75.0%	60	Alertness	AOT - incompatible stimuli	1,5	-0.15 (-1.13, 0.83)	0.90
Asaoka 2010, cognitive task 3	8	24±1.1	75.0%	60	Cognitive performance	AOT - compatible stimuli	1,5	-0.02 (-1.00, 0.96)	0.90
Asaoka 2010, cognitive task 4	8	24±1.1	75.0%	60	Cognitive performance	AOT - incompatible stimuli	1,5	0.13 (-0.85, 1.11)	0.90
Bauckhaus 2006, alertness task	34	20.3±0.4	-	60	Alertness	TAP	15	-0.07 (-0.74, 0.60)	1.44
Bauckhaus 2006, declarative memory task	34	20.3±0.4	-	60	Memory	PAL	15	0.88 (0.17, 1.58)	1.37
Bauckhaus 2006, procedural memory task	34	20.3±0.4	-	60	Cognitive performance	MTT	15	0.70 (0.01, 1.40)	1.39
Bauckhaus 2006, working memory task	34	20.3±0.4	-	60	Memory	DSB	15	0.22 (-0.46, 0.89)	1.43
Debarnot 2011, memory task 1 long nap	17	26±4.4	-	90	Memory	Sequential finger-tapping task	30	0.94 (0.06, 1.83)	1.04
Debarnot 2011, memory task 2 long nap	19	26±4.4	-	90	Memory	Sequential finger-tapping task	30	0.89 (0.01, 1.77)	1.04
Debarnot 2011, memory task 3 long nap	17	26±4.4	-	90	Memory	Sequential finger-tapping task	30	0.08 (-0.75, 0.92)	1.12
Debarnot 2011, memory task 4 long nap	19	26±4.4	-	90	Memory	Sequential finger-tapping task	30	0.08 (-0.76, 0.91)	1.12
Debarnot 2011, memory task 5 long nap	17	26±4.4	-	90	Memory	Sequential finger-tapping task	30	0.91 (0.03, 1.79)	1.04
Debarnot 2011, memory task 6 long nap	19	26±4.4	-	90	Memory	Sequential finger-tapping task	30	0.86 (-0.01, 1.74)	1.05
Debarnot 2011, memory task 7 long nap	17	26±4.4	-	90	Memory	Sequential finger-tapping task	30	0.24 (-0.60, 1.08)	1.11
Debarnot 2011, memory task 8 long nap	19	26±4.4	-	90	Memory	Sequential finger-tapping task	30	0.23 (-0.61, 1.07)	1.11
Milner 2006, reaction time task 1	34	20.2±2.27	-	20	Alertness	Ball and cup test	30	0.37 (-0.31, 1.05)	1.43
Milner 2006, reaction time task 2	34	20.2±2.27	-	20	Alertness	Ball and cup test	30	0.19 (-0.48, 0.86)	1.44
Takahashi 2004, perceived alertness 1	8	49	-	15	Alertness	Alertness scale	30	-0.36 (-1.34, 0.63)	0.89
Takahashi 2004, perceived alertness 10	8	49	-	15	Alertness	Alertness scale	30	0.60 (-0.41, 1.60)	0.87
Takahashi 2004, perceived alertness 2	8	49	-	15	Alertness	Alertness scale	30	-0.69 (-1.70, 0.32)	0.86
Takahashi 2004, perceived alertness 3	8	49	-	15	Alertness	Alertness scale	30	0.17 (-0.81, 1.15)	0.90
Takahashi 2004, perceived alertness 4	8	49	-	15	Alertness	Alertness scale	30	-0.40 (-1.39, 0.59)	0.89
Takahashi 2004, perceived alertness 5	8	49	-	15	Alertness	Alertness scale	30	0,00 (-0.98, 0.98)	0.90
Takahashi 2004, perceived alertness 6	8	49	-	15	Alertness	Alertness scale	30	0.37 (-0.62, 1.36)	0.89
Takahashi 2004, perceived alertness 7	8	49	-	15	Alertness	Alertness scale	30	0.97 (-0.07, 2.02)	0.82
Takahashi 2004, perceived alertness 8	8	49	-	15	Alertness	Alertness scale	30	0.54 (-0.46, 1.53)	0.88
Takahashi 2004, perceived alertness 9	8	49	-	15	Alertness	Alertness scale	30	0.34 (-0.64, 1.33)	0.89
**Sub total**, p= 0.205 (I squared = 16.6%)								**0.39 (0.23, 0.56)**	35.09
**31 to 60 minutes**									
Bauckhaus 2016, declarative task 1 long nap	21	70.4±6.1	45.2%	90	Memory	VLMT	60	0.17 (-0.71, 1.05)	1.05
Bauckhaus 2016, declarative task 2 long nap	21	70.4±6.1	45.2%	90	Memory	VLMT	60	0.09 (-0.78, 0.97)	1.05
Bauckhaus 2016, declarative task 3 long nap	21	70.4±6.1	45.2%	90	Memory	VLMT	60	0.16 (-0.71, 1.04)	1.05
Bauckhaus 2016, declarative task 4 long nap	21	70.4±6.1	45.2%	90	Memory	VLMT	60	0.23 (-0.65, 1.11)	1.04
Bauckhaus 2016, declarative task 5 long nap	21	70.4±6.1	45.2%	90	Memory	VLMT	60	0.13 (-0.74, 1.01)	1.05
Bauckhaus 2016, memory task 1 long nap	20	70.4±6.1	45.2%	90	Memory	MAT	35	1.39 (0.40, 2.37)	0.89
Bauckhaus 2016, memory task 2 long nap	20	70.4±6.1	45.2%	90	Memory	MAT	35	1.03 (0.09, 1.97)	0.96
Bauckhaus 2016, memory task 3 long nap	20	70.4±6.1	45.2%	90	Memory	MAT	35	1.23 (0.26, 2.19)	0.92
Bauckhaus 2016, memory task 4 long nap	20	70.4±6.1	45.2%	90	Memory	MAT	35	0.89 (-0.03, 1.82)	0.98
Bauckhaus 2016, memory task 5 long nap	20	70.4±6.1	45.2%	90	Memory	MAT	35	0.95 (0.02, 1.88)	0.97
Milner 2006, auditory oddball task 1	35	20.2±2.27	-	20	Alertness	Auditory oddball task	50	0.27 (-0.39, 0.93)	1.48
Milner 2006, auditory oddball task 2	35	20.2±2.27	-	20	Alertness	Auditory oddball task	50	0.11 (-0.54, 0.76)	1.48
Milner 2006, auditory oddball task 3	35	20.2±2.27	-	20	Alertness	Auditory oddball task	50	-0.28 (-0.93, 0.38)	1.48
Milner 2006, auditory oddball task 4	35	20.2±2.27	-	20	Alertness	Auditory oddball task	50	-0.19 (-0.85, 0.46)	1.48
Milner 2006, auditory oddball task 5	35	20.2±2.27	-	20	Alertness	Auditory oddball task	50	0.30 (-0.36, 0.95)	1.47
**Sub total**, p= 0.130 (I squared = 30.0%)								**0.35 (0.10, 0.60)**	17.34
**61 to 120 minutes**									
Bauckhaus 2016, declarative task 1 short nap	23	70.4±6.1	45.2%	45	Memory	VLMT	105	0.28 (-0.53, 1.08)	1.17
Bauckhaus 2016, declarative task 2 short nap	23	70.4±6.1	45.2%	45	Memory	VLMT	105	0.55 (-0.27, 1.36)	1.15
Bauckhaus 2016, declarative task 3 short nap	23	70.4±6.1	45.2%	45	Memory	VLMT	105	0.14 (-0.66, 0.94)	1.18
Bauckhaus 2016, declarative task 4 short nap	23	70.4±6.1	45.2%	45	Memory	VLMT	105	0.03 (-0.77, 0.83)	1.18
Bauckhaus 2016, declarative task 5 short nap	23	70.4±6.1	45.2%	45	Memory	VLMT	105	0.16 (-0.64, 0.96)	1.18
Bauckhaus 2016, memory task 1 short nap	20	70.4±6.1	45.2%	45	Memory	MAT	80	1.57 (0.56, 2.59)	0.86
Bauckhaus 2016, memory task 2 short nap	20	70.4±6.1	45.2%	45	Memory	MAT	80	1.10 (0.15, 2.05)	0.94
Bauckhaus 2016, memory task 3 short nap	20	70.4±6.1	45.2%	45	Memory	MAT	80	1.09 (0.15, 2.04)	0.95
Bauckhaus 2016, memory task 4 short nap	20	70.4±6.1	45.2%	45	Memory	MAT	80	0.68 (-0.22, 1.59)	**1.01**
Bauckhaus 2016, memory task 5 short nap	20	70.4±6.1	45.2%	45	Memory	MAT	80	0.72 (-0.19, 1.62)	1,00
Debarnot 2011, memory task 1 short nap	16	26±4.4	-	20	Memory	Sequential finger-tapping task	100	1.38 (0.40, 2.37)	0.89
Debarnot 2011, memory task 2 short nap	18	26±4.4	-	20	Memory	Sequential finger-tapping task	100	1.30 (0.33, 2.28)	0.91
Debarnot 2011, memory task 3 short nap	16	26±4.4	-	20	Memory	Sequential finger-tapping task	100	0.30 (-0.59, 1.18)	1.04
Debarnot 2011, memory task 4 short nap	18	26±4.4	-	20	Memory	Sequential finger-tapping task	100	0.28 (-0.60, 1.16)	1.04
Debarnot 2011, memory task 5 short nap	16	26±4.4	-	20	Memory	Sequential finger-tapping task	100	1.82 (0.76, 2.88)	0.81
Debarnot 2011, memory task 6 short nap	18	26±4.4	-	20	Memory	Sequential finger-tapping task	100	1.71 (0.67, 2.75)	0.83
Debarnot 2011, memory task 7 short nap	16	26±4.4	-	20	Memory	Sequential finger-tapping task	100	0.33 (-0.55, 1.21)	1.04
Debarnot 2011, memory task 8 short nap	18	26±4.4	-	20	Memory	Sequential finger-tapping task	100	0.31 (-0.57, 1.19)	1.04
Van Schalkwijk 2017,declarative memory task	40	23.3±2.4	52.6%	90	Memory	WPT	65	-0.32 (-0.98, 0.34)	1.47
Van Schalkwijk 2017,procedural memory task	36	23.3±2.4	52.6%	90	Cognitive performance	MTT	65	0.65 (-0.04, 1.34)	1.40
**Sub total**, p= 0.019 (I squared = 44.0%)								**0.64 (0.38, 0.90)**	21.07
**More than 120 minutes**									
Lau 2010, associative inference task 1	31	21.2±1.1	50.0%	90	Memory	Direct associative (face-object) memory (AB pairs)	125	-0.33 (-1.07, 0.42)	1.28
Lau 2010, associative inference task 2	31	21.2±1.1	50.0%	90	Memory	Direct associative (face-object) memory (BC pairs)	125	-0.24 (-0.98, 0.51)	1.29
Lau 2010, associative inference task 3	31	21.2±1.1	50.0%	90	Memory	Direct associative (face-object) memory (AB + BC pairs)	125	-0.31 (-1.05, 0.44)	1.28
Takahashi 2004, perceived alertness 1	8	49	-	15	Alertness	Alertness scale	135	-0.69 (-1.70, 0.33)	0.86
Takahashi 2004, perceived alertness 10	8	49	-	15	Alertness	Alertness scale	135	0.56 (-0.44, 1.56)	0.87
Takahashi 2004, perceived alertness 2	8	49	-	15	Alertness	Alertness scale	135	-1.65 (-2.81, -0.50)	0.71
Takahashi 2004, perceived alertness 3	8	49	-	15	Alertness	Alertness scale	135	-0.14 (-1.12, 0.84)	0.90
Takahashi 2004, perceived alertness 4	8	49	-	15	Alertness	Alertness scale	135	-0.60 (-1.60, 0.41)	0.87
Takahashi 2004, perceived alertness 5	8	49	-	15	Alertness	Alertness scale	135	-0.38 (-1.37, 0.61)	0.89
Takahashi 2004, perceived alertness 6	8	49	-	15	Alertness	Alertness scale	135	-0.08 (-1.06, 0.90)	0.90
Takahashi 2004, perceived alertness 7	8	49	-	15	Alertness	Alertness scale	135	0.45 (-0.55, 1.44)	0.88
Takahashi 2004, perceived alertness 8	8	49	-	15	Alertness	Alertness scale	135	-0.08 (-1.06, 0.90)	0.90
Takahashi 2004, perceived alertness 9	8	49	-	15	Alertness	Alertness scale	135	0.34 (-0.64, 1.33)	0.89
Takahashi 2004, reaction time task 1	8	49	-	15	Alertness	Choice reaction time	135	0.34 (-0.65, 1.33)	0.89
Takahashi 2004, reaction time task 2	8	49	-	15	Alertness	Choice reaction time	135	0.28 (-0.70, 1.27)	0.89
Takahashi 2004, reaction time task 3	8	49	-	15	Alertness	Choice reaction time	135	-0.19 (-1.17, 0.79)	0.90
Takahashi 2004, reaction time task 4	8	49	-	15	Alertness	Choice reaction time	135	0.16 (-0.83, 1.14)	0.90
Takahashi 2004, reaction time task 5	8	49	-	15	Alertness	Choice reaction time	135	0.18 (-0.80, 1.16)	0.90
Takahashi 2004, reaction time task 6	8	49	-	15	Alertness	Choice reaction time	135	-0.70 (-1.71, 0.32)	0.86
Tucker 2006, digit span	29	20.1	50.0%	60	Memory	DSB	240	0.20 (-0.60, 1.00)	1.17
Tucker 2006, mirror tracing 1	29	20.1	50.0%	60	Cognitive performance	MTT	240	0.62 (-0.20, 1.44)	1.14
Tucker 2006, mirror tracing 2	29	20.1	50.0%	60	Cognitive performance	MTT	240	-0.36 (-1.17, 0.44)	1.17
Tucker 2006, paired associates	29	20.1	50.0%	60	Memory	Paired associates task	240	0.75 (-0.08, 1.58)	1.13
Tucker 2008, cognitive task 1	33	23.3	-	60	Cognitive performance	Maze learning task	125	0.88 (0.16, 1.61)	1.32
Tucker 2008, cognitive task 2	33	23.3	-	60	Cognitive performance	Maze learning task	125	0.84 (0.12, 1.57)	1.32
Tucker 2008, memory task	33	23.3	-	60	Memory	SUPA	125	0.23 (-0.46, 0.93)	1.39
**Sub total**, p= 0.074 (I squared = 30.2%)								**0.05 (-0.16, 0.26)**	26.5
**Overall**, p = 0.000 (I squared = 35.8%)								**0.34 (0.23, 0.45)**	100,0

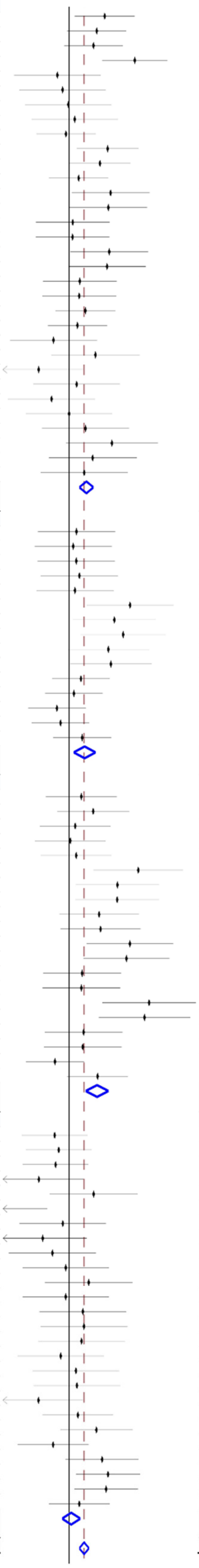

Figure S4. Summary of meta-analyses on cognitive performance at t1 compared with baseline (t0) within the nap group, stratified on type of cognitive function and on time of analysis:

Each summary of meta-analysis is presented in three conditions: global model with all the studies, after exclusion of outliers (studies not evenly distributed around the funnel plot) and with only randomized controlled trials

Each summary of several meta-analysis is represented in the forest-plot by a dot on a horizontal line. The black dots represent the pooled-effect estimate (pooled effect size - ES), and the length of each line around the dots represent their 95% confidence interval (95CI). Shorter lines represent a narrower 95CI thus higher precision around pooled-ES. Conversely, longer lines represent a wider 95CI and less precision around pooled-ES. An overall summary of the results of the meta-analyses pooled-estimate (result of the overall meta-analysis) is represented by a blue lozenge at the end of the graph. The black solid vertical line represents the null estimate (with a value of 0 for pooled-ES). Horizontal lines that cross the null vertical line represent the non-significant overall summary of the meta-analysis.

		**n studies** (subgroups)	**I-squared** (%)	**Effect size** (95% CI)	**Weight** (%)
**By type of performance**					
**Global model**	Memory	7 (45)	34.8	0.50 (0.35 to 0.66)	48.6
	Alertness	5 (38)	26.0	0.09 (-0.08 to 0.26)	39.0
	Executive functions	6 (10)	0.0	0.53 (0.29 to 0.76)	12.4
	**Overall**	11 (93)	35.8	**0.34 (0.23 to 0.45)**	100
**After exclusion of outliers**	Memory	6 (42)	19.8	0.48 (0.33 to 0.62)	48.3
	Alertness	5 (33)	0.0	0.11 (-0.03 to 0.26)	37.7
	Executive functions	6 (10)	0.0	0.53 (0.29 to 0.76)	14.0
	**Overall**	11 (85)	12.9	**0.34 (0.25 to 0.44)**	100
**only RCT**	Memory	5 (42)	38.0	0.52 (0.35 to 0.66)	75.4
	Alertness	2 (8)	0.0	0.08 (-0.15 to 0.32)	19.9
	Executive functions	2 (2)	0.0	0.68 (0.19 to 1.16)	4.7
	**Overall**	6 (52)	35.7	**0.44 (0.29 to 0.58)**	100
**By time**					
**Global model**	Less than 30 min	6 (32)	16.6	0.39 (0.23 to 0.56)	35.1
	31 to 60 min	2 (15)	30.0	0.35 (0.10 to 0.60)	17.3
	61 to 120 min	3 (20)	44.0	0.64 (0.38 to 0.90)	21.1
	>120 min	4 (26)	30.2	0.05 (-0.16 to 0.26)	26.5
	**Overall**	11 (93)	35.8	**0.34 (0.23 to 0.45)**	100
**After exclusion of outliers**	Less than 30 min	6 (30)	0.0	0.38 (0.23 to 0.53)	35.7
	31 to 60 min	2 (15)	30.0	0.35 (0.10 to 0.60)	19.1
	61 to 120 min	3 (17)	10.1	0.59 (0.37 to 0.81)	18.9
	>120 min	4 (24)	6.8	0.13 (-0.06 to 0.31)	26.3
	**Overall**	11 (86)	12.9	**0.34 (0.25 to 0.44)**	100
**only RCT**	Less than 30 min	3 (14)	0.0	0.43 (0.23 to 0.64)	28.9
	31 to 60 min	2 (15)	30.0	0.35 (0.10 to 0.60)	29.2
	61 to 120 min	3 (20)	44.0	0.64 (0.38 to 0.90)	35.4
	>120 min	1 (3)	0.0	-0.29 (-0.72 to 0.14)	6.5
	**Overall**	6 (52)	35.7	**0.44 (0.29 to 0.58)**	100

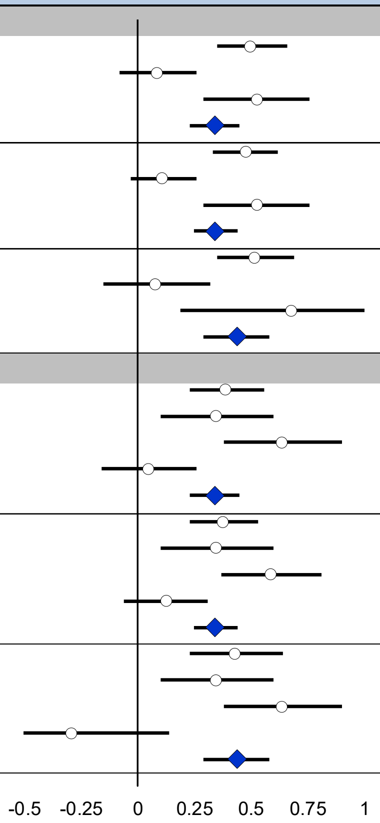

Figure S5. Meta-analysis on overall effects of nap within the nap group (t1 vs t0) stratified on type of cognitive function

95%CI: 95% confidence intervals ; AOT, Arrow orientation task; CPT II, Conner's Continuous Performance Test Version 5; DSB, Digit span backwards task; EEG, Electroencephalographic; MAT, Motor adaptation task; MTT, Mirror Tracing Task; PAL, Paired associates learning; SUPA, Semantically unrelated paired associates; TAP, Test of Attentional Performance; VLMT, Verbal learning and memory test; WPT, Word-pair task.

Each individual study or subgroup used in the meta-analysis is represented in the forest-plot by a dot on a horizontal line. The black dots represent the effect estimate (effect size - ES), and the length of each line around the dots represent its 95% confidence interval (95CI). Shorter lines represent a narrower 95CI thus higher precision around study ES, usually found in larger studies. Conversely, longer lines represent a wider 95CI and less precision around ES usually found in smaller studies. An overall summary pooled-estimate (result of the meta-analysis) is represented by a blue lozenge at the end of the graph. The black solid vertical line represents the null estimate (with a value of 0 for ES) and the red dotted vertical line represents result of the meta-analysis (pooled-estimate). Horizontal lines that cross the null vertical line represent non-significant studies.

Study	n	Age, years (mean±SD)	Sex (% male)	Nap opportunity (min)	Type of cognitive function	Type of test	Time between nap & test (min)	Effect size (95CI)	Weight %
**Memory**									
Bauckhaus 2006, declarative memory task	34	20.3±0.4	-	60	Memory	PAL	15	0.88 (0.17, 1.58)	1.37
Bauckhaus 2006, working memory task	34	20.3±0.4	-	60	Memory	DSB	15	0.22 (-0.46, 0.89)	1.43
Bauckhaus 2016, declarative task 1 long nap	21	70.4±6.1	45.2%	90	Memory	VLMT	60	0.17 (-0.71, 1.05)	1.05
Bauckhaus 2016, declarative task 1 short nap	23	70.4±6.1	45.2%	45	Memory	VLMT	105	0.28 (-0.53, 1.08)	1.17
Bauckhaus 2016, declarative task 2 long nap	21	70.4±6.1	45.2%	90	Memory	VLMT	60	0.09 (-0.78, 0.97)	1.05
Bauckhaus 2016, declarative task 2 short nap	23	70.4±6.1	45.2%	45	Memory	VLMT	105	0.55 (-0.27, 1.36)	1.15
Bauckhaus 2016, declarative task 3 long nap	21	70.4±6.1	45.2%	90	Memory	VLMT	60	0.16 (-0.71, 1.04)	1.05
Bauckhaus 2016, declarative task 3 short nap	23	70.4±6.1	45.2%	45	Memory	VLMT	105	0.14 (-0.66, 0.94)	1.18
Bauckhaus 2016, declarative task 4 long nap	21	70.4±6.1	45.2%	90	Memory	VLMT	60	0.23 (-0.65, 1.11)	1.04
Bauckhaus 2016, declarative task 4 short nap	23	70.4±6.1	45.2%	45	Memory	VLMT	105	0.03 (-0.77, 0.83)	1.18
Bauckhaus 2016, declarative task 5 long nap	21	70.4±6.1	45.2%	90	Memory	VLMT	60	0.13 (-0.74, 1.01)	1.05
Bauckhaus 2016, declarative task 5 short nap	23	70.4±6.1	45.2%	45	Memory	VLMT	105	0.16 (-0.64, 0.96)	1.18
Bauckhaus 2016, memory task 1 long nap	20	70.4±6.1	45.2%	90	Memory	MAT	35	1.39 (0.40, 2.37)	0.89
Bauckhaus 2016, memory task 1 short nap	20	70.4±6.1	45.2%	45	Memory	MAT	80	1.57 (0.56, 2.59)	0.86
Bauckhaus 2016, memory task 2 long nap	20	70.4±6.1	45.2%	90	Memory	MAT	35	1.03 (0.09, 1.97)	0.96
Bauckhaus 2016, memory task 2 short nap	20	70.4±6.1	45.2%	45	Memory	MAT	80	1.10 (0.15, 2.05)	0.94
Bauckhaus 2016, memory task 3 long nap	20	70.4±6.1	45.2%	90	Memory	MAT	35	1.23 (0.26, 2.19)	0.92
Bauckhaus 2016, memory task 3 short nap	20	70.4±6.1	45.2%	45	Memory	MAT	80	1.09 (0.15, 2.04)	0.95
Bauckhaus 2016, memory task 4 long nap	20	70.4±6.1	45.2%	90	Memory	MAT	35	0.89 (-0.03, 1.82)	0.98
Bauckhaus 2016, memory task 4 short nap	20	70.4±6.1	45.2%	45	Memory	MAT	80	0.68 (-0.22, 1.59)	**1.01**
Bauckhaus 2016, memory task 5 long nap	20	70.4±6.1	45.2%	90	Memory	MAT	35	0.95 (0.02, 1.88)	0.97
Bauckhaus 2016, memory task 5 short nap	20	70.4±6.1	45.2%	45	Memory	MAT	80	0.72 (-0.19, 1.62)	1,00
Debarnot 2011, memory task 1 long nap	17	26±4.4	-	90	Memory	Sequential finger-tapping task	30	0.94 (0.06, 1.83)	1.04
Debarnot 2011, memory task 1 short nap	16	26±4.4	-	20	Memory	Sequential finger-tapping task	100	1.38 (0.40, 2.37)	0.89
Debarnot 2011, memory task 2 long nap	19	26±4.4	-	90	Memory	Sequential finger-tapping task	30	0.89 (0.01, 1.77)	1.04
Debarnot 2011, memory task 2 short nap	18	26±4.4	-	20	Memory	Sequential finger-tapping task	100	1.30 (0.33, 2.28)	0.91
Debarnot 2011, memory task 3 long nap	17	26±4.4	-	90	Memory	Sequential finger-tapping task	30	0.08 (-0.75, 0.92)	1.12
Debarnot 2011, memory task 3 short nap	16	26±4.4	-	20	Memory	Sequential finger-tapping task	100	0.30 (-0.59, 1.18)	1.04
Debarnot 2011, memory task 4 long nap	19	26±4.4	-	90	Memory	Sequential finger-tapping task	30	0.08 (-0.76, 0.91)	1.12
Debarnot 2011, memory task 4 short nap	18	26±4.4	-	20	Memory	Sequential finger-tapping task	100	0.28 (-0.60, 1.16)	1.04
Debarnot 2011, memory task 5 long nap	17	26±4.4	-	90	Memory	Sequential finger-tapping task	30	0.91 (0.03, 1.79)	1.04
Debarnot 2011, memory task 5 short nap	16	26±4.4	-	20	Memory	Sequential finger-tapping task	100	1.82 (0.76, 2.88)	0.81
Debarnot 2011, memory task 6 long nap	19	26±4.4	-	90	Memory	Sequential finger-tapping task	30	0.86 (-0.01, 1.74)	1.05
Debarnot 2011, memory task 6 short nap	18	26±4.4	-	20	Memory	Sequential finger-tapping task	100	1.71 (0.67, 2.75)	0.83
Debarnot 2011, memory task 7 long nap	17	26±4.4	-	90	Memory	Sequential finger-tapping task	30	0.24 (-0.60, 1.08)	1.11
Debarnot 2011, memory task 7 short nap	16	26±4.4	-	20	Memory	Sequential finger-tapping task	100	0.33 (-0.55, 1.21)	1.04
Debarnot 2011, memory task 8 long nap	19	26±4.4	-	90	Memory	Sequential finger-tapping task	30	0.23 (-0.61, 1.07)	1.11
Debarnot 2011, memory task 8 short nap	18	26±4.4	-	20	Memory	Sequential finger-tapping task	100	0.31 (-0.57, 1.19)	1.04
Lau 2010, associative inference task 1	31	21.2±1.1	50.0%	90	Memory	Direct associative (face-object) memory (AB pairs)	125	-0.33 (-1.07, 0.42)	1.28
Lau 2010, associative inference task 2	31	21.2±1.1	50.0%	90	Memory	Direct associative (face-object) memory (BC pairs)	125	-0.24 (-0.98, 0.51)	1.29
Lau 2010, associative inference task 3	31	21.2±1.1	50.0%	90	Memory	Direct associative (face-object) memory (AB + BC pairs)	125	-0.31 (-1.05, 0.44)	1.28
Tucker 2006, digit span	29	20.1	50.0%	60	Memory	DSB	240	0.20 (-0.60, 1.00)	1.17
Tucker 2006, paired associates	29	20.1	50.0%	60	Memory	Paired associates task	240	0.75 (-0.08, 1.58)	1.13
Tucker 2008, memory task	33	23.3	-	60	Memory	SUPA	125	0.23 (-0.46, 0.93)	1.39
Van Schalkwijk 2017,declarative memory task	40	23.3±2.4	52.6%	90	Memory	WPT	65	-0.32 (-0.98, 0.34)	1.47
**Sub total**, p=0.939 (I squared = 0.0%)								**0.50 (0.35, 0.66)**	48.58
**Alertness**									
Amin 2012, cognitive task 1	29	29±1.9	61.0%	20	Alertness	CPT II	25	0.81 (0.13, 1.49)	1.42
Amin 2012, EEG recording	29	29±1.9	61.0%	20	Alertness	EEG recording	25	1.49 (0.75, 2.24)	1.29
Asaoka 2010, cognitive task 1	8	24±1.1	75.0%	60	Alertness	AOT - compatible stimuli	1,5	-0.27 (-1.25, 0.72)	0.89
Asaoka 2010, cognitive task 2	8	24±1.1	75.0%	60	Alertness	AOT - incompatible stimuli	1,5	-0.15 (-1.13, 0.83)	0.9
Bauckhaus 2006, alertness task	34	20.3±0.4	-	60	Alertness	TAP	15	-0.07 (-0.74, 0.60)	1.44
Milner 2006, auditory oddball task 1	35	20.2±2.27	-	20	Alertness	Auditory oddball task	50	0.27 (-0.39, 0.93)	1.48
Milner 2006, auditory oddball task 2	35	20.2±2.27	-	20	Alertness	Auditory oddball task	50	0.11 (-0.54, 0.76)	1.48
Milner 2006, auditory oddball task 3	35	20.2±2.27	-	20	Alertness	Auditory oddball task	50	-0.28 (-0.93, 0.38)	1.48
Milner 2006, auditory oddball task 4	35	20.2±2.27	-	20	Alertness	Auditory oddball task	50	-0.19 (-0.85, 0.46)	1.48
Milner 2006, auditory oddball task 5	35	20.2±2.27	-	20	Alertness	Auditory oddball task	50	0.30 (-0.36, 0.95)	1.47
Milner 2006, reaction time task 1	34	20.2±2.27	-	20	Alertness	Ball and cup test	30	0.37 (-0.31, 1.05)	1.43
Milner 2006, reaction time task 2	34	20.2±2.27	-	20	Alertness	Ball and cup test	30	0.19 (-0.48, 0.86)	1.44
Takahashi 2004, perceived alertness 1	8	49	-	15	Alertness	Alertness scale	30	-0.36 (-1.34, 0.63)	0.89
Takahashi 2004, perceived alertness 1	8	49	-	15	Alertness	Alertness scale	135	-0.69 (-1.70, 0.33)	0.86
Takahashi 2004, perceived alertness 10	8	49	-	15	Alertness	Alertness scale	30	0.60 (-0.41, 1.60)	0.87
Takahashi 2004, perceived alertness 10	8	49	-	15	Alertness	Alertness scale	135	0.56 (-0.44, 1.56)	0.87
Takahashi 2004, perceived alertness 2	8	49	-	15	Alertness	Alertness scale	30	-0.69 (-1.70, 0.32)	0.86
Takahashi 2004, perceived alertness 2	8	49	-	15	Alertness	Alertness scale	135	-1.65 (-2.81, -0.50)	0.71
Takahashi 2004, perceived alertness 3	8	49	-	15	Alertness	Alertness scale	30	0.17 (-0.81, 1.15)	0.9
Takahashi 2004, perceived alertness 3	8	49	-	15	Alertness	Alertness scale	135	-0.14 (-1.12, 0.84)	0.9
Takahashi 2004, perceived alertness 4	8	49	-	15	Alertness	Alertness scale	30	-0.40 (-1.39, 0.59)	0.89
Takahashi 2004, perceived alertness 4	8	49	-	15	Alertness	Alertness scale	135	-0.60 (-1.60, 0.41)	0.87
Takahashi 2004, perceived alertness 5	8	49	-	15	Alertness	Alertness scale	30	0,00 (-0.98, 0.98)	0.9
Takahashi 2004, perceived alertness 5	8	49	-	15	Alertness	Alertness scale	135	-0.38 (-1.37, 0.61)	0.89
Takahashi 2004, perceived alertness 6	8	49	-	15	Alertness	Alertness scale	30	0.37 (-0.62, 1.36)	0.89
Takahashi 2004, perceived alertness 6	8	49	-	15	Alertness	Alertness scale	135	-0.08 (-1.06, 0.90)	0.9
Takahashi 2004, perceived alertness 7	8	49	-	15	Alertness	Alertness scale	30	0.97 (-0.07, 2.02)	0.82
Takahashi 2004, perceived alertness 7	8	49	-	15	Alertness	Alertness scale	135	0.45 (-0.55, 1.44)	0.88
Takahashi 2004, perceived alertness 8	8	49	-	15	Alertness	Alertness scale	30	0.54 (-0.46, 1.53)	0.88
Takahashi 2004, perceived alertness 8	8	49	-	15	Alertness	Alertness scale	135	-0.08 (-1.06, 0.90)	0.9
Takahashi 2004, perceived alertness 9	8	49	-	15	Alertness	Alertness scale	30	0.31 (-0.67, 1.30)	0.89
Takahashi 2004, perceived alertness 9	8	49	-	15	Alertness	Alertness scale	135	0.34 (-0.64, 1.33)	0.89
Takahashi 2004, reaction time task 1	8	49	-	15	Alertness	Choice reaction time	135	0.34 (-0.65, 1.33)	0.89
Takahashi 2004, reaction time task 2	8	49	-	15	Alertness	Choice reaction time	135	0.28 (-0.70, 1.27)	0.89
Takahashi 2004, reaction time task 3	8	49	-	15	Alertness	Choice reaction time	135	-0.19 (-1.17, 0.79)	0.9
Takahashi 2004, reaction time task 4	8	49	-	15	Alertness	Choice reaction time	135	0.16 (-0.83, 1.14)	0.9
Takahashi 2004, reaction time task 5	8	49	-	15	Alertness	Choice reaction time	135	0.18 (-0.80, 1.16)	0.9
Takahashi 2004, reaction time task 6	8	49	-	15	Alertness	Choice reaction time	135	-0.70 (-1.71, 0.32)	0.86
**Sub total**, p=0.009 (I squared = 38.9%)								**0.09 (-0.08, 0.26)**	38.98
**Executive functions**									
Amin 2012, cognitive task 2	29	29±1.9	61.0%	20	Cognitive performance	CPT II	25	0.63 (-0.04, 1.30)	1.44
Amin 2012, cognitive task 3	29	29±1.9	61.0%	20	Cognitive performance	CPT II	25	0.55 (-0.11, 1.22)	1.45
Asaoka 2010, cognitive task 3	8	24±1.1	75.0%	60	Cognitive performance	AOT - compatible stimuli	1,5	-0.02 (-1.00, 0.96)	0.9
Asaoka 2010, cognitive task 4	8	24±1.1	75.0%	60	Cognitive performance	AOT - incompatible stimuli	1,5	0.13 (-0.85, 1.11)	0.9
Bauckhaus 2006, procedural memory task	34	20.3±0.4	-	60	Cognitive performance	MTT	15	0.70 (0.01, 1.40)	1.39
Tucker 2006, mirror tracing 1	29	20.1	50.0%	60	Cognitive performance	MTT	240	0.62 (-0.20, 1.44)	1.14
Tucker 2006, mirror tracing 2	29	20.1	50.0%	60	Cognitive performance	MTT	240	-0.36 (-1.17, 0.44)	1.17
Tucker 2008, cognitive task 1	33	23.3	-	60	Cognitive performance	Maze learning task	125	0.88 (0.16, 1.61)	1.32
Tucker 2008, cognitive task 2	33	23.3	-	60	Cognitive performance	Maze learning task	125	0.84 (0.12, 1.57)	1.32
Van Schalkwijk 2017,procedural memory task	36	23.3±2.4	52.6%	90	Cognitive performance	MTT	65	0.65 (-0.04, 1.34)	1.4
**Sub total**, p=0.949 (I squared = 0.0%)								**0.53 (-0.29, 0.76)**	12.44
**Overall**, p = 0.000 (I squared = 35.8%)								**0.34 (0.23, 0.45)**	100.00

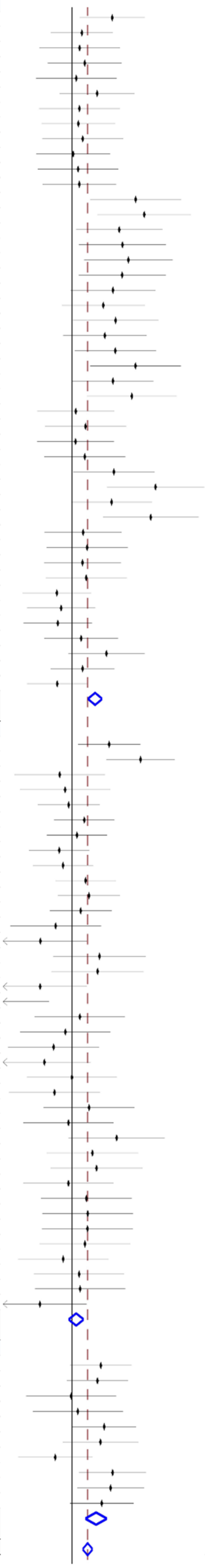

Figure S6. Summary of meta-analyses on changes in performance between t1 and t0 between groups

Each summary of meta-analysis is presented in three conditions: global model with all the studies, after exclusion of outliers (studies not evenly distributed around the funnel plot) and with only randomized controlled trials

Each summary of several meta-analysis is represented in the forest-plot by a dot on a horizontal line. The black dots represent the pooled-effect estimate (pooled effect size - ES), and the length of each line around the dots represent their 95% confidence interval (95CI). Shorter lines represent a narrower 95CI thus higher precision around pooled-ES. Conversely, longer lines represent a wider 95CI and less precision around pooled-ES. An overall summary of the results of the meta-analyses pooled-estimate (result of the overall meta-analysis) is represented by a blue lozenge at the end of the graph. The black solid vertical line represents the null estimate (with a value of 0 for pooled-ES). Horizontal lines that cross the null vertical line represent the non-significant overall summary of the meta-analysis.

		n studies (subgroups)	I-squared (%)	Effect size (95% CI)	Weight (%)
**By type of performance**					
**Global model**	Memory	7 (45)	93.0	0.47 (-0.16 to 1.10)	52.0
	Alertness	5 (38)	93.4	2.36 (1.53 to 3.19)	37.3
	Executive functions	6 (10)	94.7	1.65 (0.23 to 3.08)	10.7
	**Overall**	11 (93)	93.3	**1.26 (0.79 to 1.73)**	100
**After exclusion of outliers**	Memory	5 (42)	93.3	0.51 (-0.17 to 1.19)	51.3
	Alertness	2 (8)	93.2	1.98 (1.17 to 2.79)	37.5
	Executive functions	2 (2)	92.3	1.06 (-0.06 to 2.18)	11.2
	**Overall**	6 (52)	93.0	**1.10 (0.63 to 1.57)**	100
**only RCT**	Memory	6 (43)	92.9	0.35 (-0.32 to 1.01)	79.4
	Alertness	5 (36)	85.2	0.41 (-0.24 to 1.06)	18.2
	Executive functions	5 (9)	98.5	43.7 (-42.4 to 129.9)	2.4
	**Overall**	10 (88)	92.7	**0.38 (-0.15 to 0.92)**	100
**By time**					
**Global model**	Less than 30 min	6 (32)	94.2	3.81 (2.82 to 4.80)	31.3
	31 to 60 min	2 (15)	87.6	-1.25 (-1.93 to -0.57)	18.7
	61 to 120 min	3 (20)	94.5	1.17 (-0.05 to 2.39)	21.2
	>120 min	4 (26)	91.2	1.17 (0.40 to 1.93)	28.9
	**Overall**	11 (93)	93.3	**1.26 (0.79 to 1.73)**	100
**After exclusion of outliers**	Less than 30 min	6 (31)	94.1	3.55 (2.57 to 4.52)	32.1
	31 to 60 min	2 (14)	88.4	-1.38 (-2.11 to 0.65)	18.1
	61 to 120 min	2 (18)	93.8	0.88 (-0.35 to 2.11)	20.6
	>120 min	4 (25)	90.9	0.97 (0.22 to 1.72)	29.2
	**Overall**	8 (88)	93.0	**1.10 (0.63 to 1.57)**	100
**only RCT**	Less than 30 min	3 (14)	92.8	2.05 (1.01 to 3.10)	27.0
	31 to 60 min	2 (15)	87.6	-1.25 (-1.93 to -0.57)	31.3
	61 to 120 min	3 (20)	94.5	1.17 (-0.05 to 2.39)	34.9
	>120 min	1 (3)	0.0	0.92 (0.49 to 1.35)	6.9
	**Overall**	6 (52)	92.7	**0.38 (-0.15 to 0.92)**	100

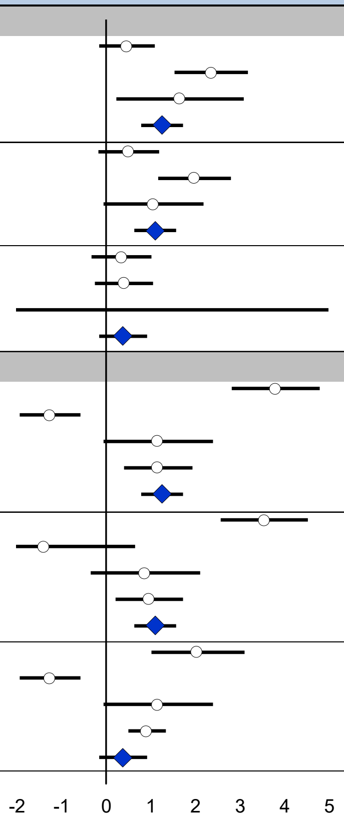

Figure S7. Summary of meta-analyses on cognitive performance at t1 compared with baseline (t0) within the control group, stratified on type of cognitive function and on time of analysis

Each summary of meta-analysis is presented in three conditions: global model with all the studies, after exclusion of outliers (studies not evenly distributed around the funnel plot) and with only randomized controlled trials

Each summary of several meta-analysis is represented in the forest-plot by a dot on a horizontal line. The black dots represent the pooled-effect estimate (pooled effect size - ES), and the length of each line around the dots represent their 95% confidence interval (95CI). Shorter lines represent a narrower 95CI thus higher precision around pooled-ES. Conversely, longer lines represent a wider 95CI and less precision around pooled-ES. An overall summary of the results of the meta-analyses pooled-estimate (result of the overall meta-analysis) is represented by a blue lozenge at the end of the graph. The black solid vertical line represents the null estimate (with a value of 0 for pooled-ES). Horizontal lines that cross the null vertical line represent the non-significant overall summary of the meta-analysis.

		n studies (subgroups)	I-squared (%)	Effect size (95% CI)	Weight (%)
**By type of performance**					
**Global model**	Memory	7 (45)	36.0	0.27 (0.10 to 0.43)	48.2
	Alertness	5 (38)	0.0	-0.15 (-0.29 to -0.01)	39.2
	Executive functions	6 (10)	60.0	0.17 (-0.21 to 0.55)	12.7
	**Overall**	11 (93)	31.8	**0.08 (-0.03 to 0.19)**	100
**After exclusion of outliers**	Memory	6 (42)	29.4	0.27 (0.11 to 0.43)	48.3
	Alertness	5 (34)	0.0	-0.13 (-0.28 to 0.02)	38.0
	Executive functions	6 (10)	60.0	0.17 (-0.21 to 0.55)	13.7
	**Overall**	11 (86)	29.9	**0.10 (-0.01 to 0.21)**	100
**only RCT**	Memory	5 (42)	37.9	0.27 (0.10 to 0.43)	75.2
	Alertness	2 (8)	0.0	0.05 (-0.18 to 0.29)	20.0
	Executive functions	2 (2)	87.6	-0.26 (-1.64 to 1.12)	4.9
	**Overall**	6 (52)	37.1	**0.20 (0.05 to 0.34)**	100
**By time**					
**Global model**	Less than 30 min	6 (32)	0.0	-0.02 (-0.18 to 0.13)	33.9
	31 to 60 min	2 (15)	8.9	0.36 (0.15 to 0.58)	17.8
	61 to 120 min	3 (20)	45.4	0.24 (-0.03 to 0.51)	20.9
	>120 min	4 (26)	48.7	-0.08 (-0.32 to 0.16)	27.5
	**Overall**	11 (93)	31.8	**0.08 (-0.03 to 0.19)**	100
**After exclusion of outliers**	Less than 30 min	6 (30)	0.0	0.00 (-0.15 to 0.16)	34.2
	31 to 60 min	2 (15)	8.9	0.36 (0.15 to 0.58)	19.2
	61 to 120 min	3 (17)	34.7	0.25 (-0.02 to 0.52)	18.9
	>120 min	4 (24)	52.4	-0.07 (-0.33 to 0.19)	27.7
	**Overall**	11 (86)	29.9	**0.10 (-0.01 to 0.21)**	100
**only RCT**	Less than 30 min	3 (14)	0.0	0.21 (-0.01 to 0.42)	27.7
	31 to 60 min	2 (15)	8.9	0.36 (0.15 to 0.58)	30.1
	61 to 120 min	3 (20)	45.4	0.24 (-0.03 to 0.51)	35.6
	>120 min	1 (3)	0.0	-0.83 (-1.26 to -0.40)	6.7
	**Overall**	6 (52)	37.1	**0.20 (0.05 to 0.34)**	100

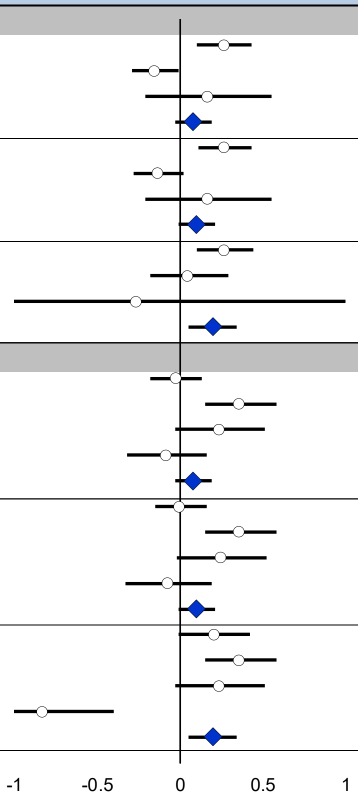

Supplement: Supplementary file 1 [file ijerph-18-10212-s001.zip › ijerph-1367211-supplementary/Supplementary Figures.pdf]
