# Supplementary material for: Effects of a Short Daytime Nap on the Cognitive Performance: A Systematic Review and Meta-Analysis
Source: Int J Environ Res Public Health. 2021 Sep 28;18(19):10212. doi: 10.3390/ijerph181910212 (PMC8507757; doi:10.3390/ijerph181910212)
Supplement: Supplementary file 1 [file ijerph-18-10212-s001.zip › ijerph-1367211-supplementary/Supplementary Materials 2.pdf]

Supplementary Materials 2. Details of test used for measure of cognitive functions in included articles.

Alertness test and condition of analyses

Electroencephalographic (EEG) and electrooculographic (EOG) recording was reported in one article.[41] It was used to detect attention failure that was defined as a 30-second wake period with one or more SEMs. SEMs consist of conjugate, reasonably regular, slow sinusoidal eye movements observed in the EOG recording. The outcome reported was the prevalence of attention failures. To determine it, the number of attention failures was divided by the total number of 30-second wake periods and the quotient was multiplied by 100 to express the result as a percentage.

Conner's Continuous Performance Test Version 5 (CPT II) was reported in one article.[41] The CPT II test asks each participant to respond to a letter flashed on a computer screen by either hitting a key or refraining from hitting a key, depending on the letter flashed. The CPT II test protocol provides a task-oriented assessment of attention problems and is widely used in research and clinical testing. Three outcomes were reported: hit reaction time, omission and commission.

Arrow-orientation task (AOT) was reported in one article.[42] AOT is a spatial stroop task. In this task, a white fixation cross was presented on a computer monitor. Then a white arrow (pointing up or down) was presented above or below fixation. Thus, stimuli consisted of two compatible and two incompatible stimuli in relation to the direction where the arrow was pointed. The task was to respond to the pointing direction of the arrow (up or down) by pressing a button with the corresponding hand. Two outcomes were reported: reaction time compatible, reaction incompatible.

Test of Attentional Performance (TAP) was reported in one article.[43] TAP is a standardized test to evaluate alertness which is published by Zimmermann and Fimm.[108] Reported outcome was the mean reaction time.

Ball and cup task was reported in one article.[46] Improvement on the ‘‘ball and cup’’ task involves implicit learning of procedural movements that are cognitively simple. Each trial consisted of an attempt to catch a ball with a cup that is attached to the ball by a string. Performance was scored for each trial on a 4-point scale (0 = miss, 1 = hit side, 2 = bounced out, 3 = catch), on 200 trials pre-condition and 200 trials post-condition. Participants were instructed to hold the ‘‘ball and cup’’ apparatus by the handle at their side and to swing the cup in an upward arc with the aim of catching the ball with the cup. Two outcomes were reported: mean of 10% fastest, number of lapses (reaction time ≥ 500ms).

Choice reaction time (RT) task was reported in one article.[47] For this test, the subjects were presented a filled circle or a filled triangle on a screen and were instructed to press a button with the thumb of their preferred hand as quickly as possible whenever they detected a filled circle. Two outcomes were reported: median response time, percentage of correct response.

Alertness scale was reported in one article.[47] Subjects rated their alertness every day using a nine-point scale in which higher values indicate greater alertness (1 = very sleepy; 9 = very alert). Mean subjective score was the outcome reported.

Executive functions test and condition of analyses

Conner’s Continuous Performance Test Version 5 (CPT II) was reported in one article.[41] It is the same that explained upper but here the two outcomes reported were omission (the number of letters to which the individual should have responded, but did not) and commission (the number of letters to which the individual should not have responded, but did).

Arrow-orientation task (AOT) was reported in one article.[42] It is the same that explained upper but here the two outcomes reported were: number of errors compatible, number of errors incompatible.

Auditory oddball task was reported in one article.[46] This task involved differentiating between tones of two different pitches. The participants' task was to respond as quickly and accurately as possible to the rare, target tones by pressing a hand-held button. The outcome reported was the reaction time corresponding to three event-related potentials (ERPs): N1, P2, and P300 measured by electroencephalography (EEG).

Mirror Tracing Task (MTT) was reported in three articles.[42,47,49] In MTT, adapted from Plihal and Born,[109] the subjects had to move an electronic stylus along the black line of a figure, which they could only see in a mirror. Three outcomes were reported: total time,[42,47] accuracy,[47] error time.[49]

Maze learning task was reported in one article.[49] This task is a computerized version of the "bolt head maze" used by Brenda Milner.[110] Subjects start at the "start" button in the lower left hand corner and move left-right or up-down clicking each square with a mouse. If a subject is on the correct path, each square lights up green. If the subject hits a "wall" the square lights up orange. With each forward mouse click the preceding square returns to its original gray color. Two outcomes were reported: number of errors, average time.

Memory test and condition of analyses

Paired associates learning (PAL) was reported in one article.[43] The PAL consisted of 40 pairs of related German nouns that were standardized with respect to word frequency, length, emotionality, meaningfulness and concreteness. The word pairs were visually presented for 5s each. Immediately after presentation of all word pairs, the subjects were asked to orally recall

the second word on presentation of the first (cued recall). Reported outcome was the number of correctly reproduced paired associates.

Digit span backwards task (DSB) was reported in two articles.[43,48] Il is a test used to assess working memory capacity. DSB consists on memorizing series of number. First, three numbers are presented. Then, each subsequent series was increased by one number until the last series of 10 numbers. Reported outcome was the number of correctly recalled number series.

Verbal learning and memory test (VLMT) was reported in one article.[44] In this test, adapted from Helmstädter,[111] participants learned lists of 15 words by repeated auditory presentation and the number of correct sequences retained was the outcome reported.

Motor adaptation task (MAT) was reported in one article.[44] To this test, on a computer targets could appear at one out of eight possible predefined locations. These predefined target locations were arranged in circular relationship to the middle of the screen and collected by moving the joystick with the left hand. Joystick movements were in turn projected as a dot-cursor on the screen. A target would disappear when the dot-cursor remained within a 12 pixel radius of the target for at least 100ms. As soon as the dot- cursor reached its neutral position in the middle of the screen, a new target would appear. The number of collected dots within the 150 s time frame was the outcome reported.

Sequential finger-tapping task was reported in one article.[45] This test, developed by Karni,[112] was used to measure motor sequence learning. Participants were first asked to memorize a sequence of eight moves employing fingers 2 to 5, using their non-dominant hand. They were requested to tap the sequence as quickly and accurately as possible on a computer keyboard. The number of correct sequences and the average speed used to perform each correct sequence were the outcomes reported.

Direct associative (face-object) memory was reported in one article.[6] It is an adapted version of an associative inference task,[113],[114] which comprised two sets of black and

white photograph pairs, each of a face and a common household object. There were 30 photograph pairs in each set. The stimuli included a total of 60 faces and 30 common objects. The sets contained an equal number of male and female face pictures, and all photographs were equated for brightness and contrast. The outcome reported was the number of correctly matched pairs.

Paired associates task was reported in one article.[48] Forty semantically related word pairs were selected from a larger pool of word pairs used by Plihal and Born (1997).[109] Subjects were shown in random order the first word of each of the 40 word pairs and asked to type in the word that completes the pair. The number of correctly completed word pairs was the outcome reported.

Sementically unrelated paired associates (SUPA) was reported in one article.[49] Sixty word pairs were created from common objects and were randomly paired to eliminate semantic relationships between the pairs. After presentation of all word pairs, subjects completed a cued recall test, during which they were presented the first word of 20 of the word and were asked to type the target word that completed the word pair. The outcome reported was the number of word pairs recalled.

Word-pair task (WPT) was reported in one article.[50] This task of 160 word-pairs applied an adapted paradigm from Plihal and Born (1997)[109] and Schabus et al., 2004,[115] 2006.[116] Word-pairs were presented twice in a blocked and randomized order. The control task instructed participants to count and verbally report the number of deviating letters within each pseudo word-pair. Percentage of correct response was the outcome reported.
