# Supplementary material for: Effects of a Short Daytime Nap on the Cognitive Performance: A Systematic Review and Meta-Analysis
Source: Int J Environ Res Public Health. 2021 Sep 28;18(19):10212. doi: 10.3390/ijerph181910212 (PMC8507757; doi:10.3390/ijerph181910212)
Supplement: Supplementary file 1 [file ijerph-18-10212-s001.zip › ijerph-1367211-supplementary/Supplementary Materials 3.pdf]

Supplementary Materials 3. Details text of sensitivity analysis

Meta-analysis on effects of nap within the nap group

Compared with baseline, cognitive performance improved at t1 within the nap group (effect size 0.34, 95% CI 0.23 to 0.45, I^2=35.8%) (Fig 6, Appendix 4 and 5). Stratified on type of cognitive functions, all types of cognitive performance increased after napping compared with controls (memory: 0.50, 0.35 to 0.66, I^2=34.8%; alertness: 0.09, -0.08 to 0.26, I^2=26.0%; executive functions: 0.53, 0.29 to 0.76, I^2=0.0%) (Appendix 3, 4 and 5). Stratified on time of analysis, cognitive performance improved less than 30 minutes (0.39, 0.23 to 0.56, I^2=16.6%), 31 to 60 minutes (0.35, 0.10 to 0.60, I^2=30.0%) and 61 to 120 minutes after nap (0.64, 0.38 to 0.90, I^2=44.0%). More than 120 minutes after nap results were non-significant (Fig 6, Appendix 3 and 4). Meta-analysis were reperformed after exclusion from metafunnels[41,44,45,47,50] (Fig 6, Appendix 3 and 4) and of non-RCT[41,42,47–49] (Fig 6, Appendix 4) and demonstrated similar results except for cognitive performance more than 120 minutes after nap which deteriorated after exclusion of non-RCT.

Meta-analysis on changes in performance between groups

Changes in performance between t1 and t0 was better in the nap group compared with controls (1.26, 0.79 to 1.73, I^2=93.3%). Stratified on type of cognitive functions, results were the same: memory (0.47, -0.16 to 1.10, I^2=93.0%), alertness (2.36, 1.53 to 3.19, I^2=93.4%) and executive functions (1.65, 0.23 to 3.08, I^2=94.7%). Stratifying analysis by time, results were noticeably the same: less than 30 minutes (3.81, 2.82 to 4.80, I^2=94.2%), between 61 and 120 minutes (1.17, -0.05 to 2.39, I^2=91.2%) and more than 120 minutes after the nap (1.17, 0.40 to 1.93, I^2=91.2%) but except between 31 and 60 minutes which deteriorated (-1.25, -1.93 to -0.57,

I^2=87.6%). Similar results were demonstrated after exclusion from metafunnels[44,47,50] and of non-RCT.[41,42,47–49] (Fig 6 and Appendix 6)

Meta-analysis on comparison of performance at t1 vs t0 within the control group

Compared with baseline, cognitive performance improved globally at t1 within the control group (0.08, -0.03 to 0.19, I^2=31.8%). Stratified on type of cognitive functions, memory and executive functions improved at t1 within the control group (0.27, 0.10 to 0.43, I^2=36.0%, and 0.17, -0.21 to 0.55, I^2=60.0% respectively), but alertness decreased (-0.15, -0.29 to -0.01, I^2=0.0%). Stratified on time of analysis, cognitive performance decreased globally less than 30 minutes (-0.02, -0.18 to 0.13, I^2=0.0%) and more than 120 minutes after rest period (without nap) (-0.08, -0.32 to 0.16, I^2=48.7%). Cognitive performance improved 31 to 60 minutes (0.36, 0.15 to 0.58, I^2=8.9%) and 61 to 120 minutes after rest period (0.24, -0.03 to 0.51, I^2=45.4%). Meta-analysis were reperformed after exclusion from metafunnels[41,44,45,47,50] and of non-RCT[41,42,47–49] and demonstrated similar results except for results less than 30 min after rest period which improved after exclusion of non-RCT. (Fig 6, Appendix 3 and 7)
