# Supplementary material for: Effects of a Short Daytime Nap on the Cognitive Performance: A Systematic Review and Meta-Analysis
Source: Int J Environ Res Public Health. 2021 Sep 28;18(19):10212. doi: 10.3390/ijerph181910212 (PMC8507757; doi:10.3390/ijerph181910212)
Supplement: Supplementary file 1 [file ijerph-18-10212-s001.zip › ijerph-1367211-supplementary/Supplementary Table S1.pdf]

Table S1. Characteristics of included studies

Study	Design			Participants						Nap		Time between nap and test	Test of cognitive performance		Quality of articles	
	Cross-over	Rando-mized	Environ-ment	Nap			Control									
				n	Age<br>mean±SD	Sex<br>% men	n	Age<br>mean±SD	Sex<br>% men	Duration<br>min	Time<br>hours	t1<br>min	Name	Function	CONSORT<br>%	STROBE<br>%
Abdessalem 2019	no	yes	Laboratory	-	21±3	-	-	21±3	-	25	13:00-13:25<br>14:00-14:25<br>15:00-15:25	80<br>140<br>200	Digit cancellation task	Alertness	-	53.1
**Amin 2012**	no	no	Real work<br>Laboratory	18	29±1.9	61	11	30±1.4	55	20	13:15-13:35	25	EEG recording<br>CPT II	Alertness<br>Cognitive performance<br>Alertness	-	50
**Asaoka 2010**	yes	no	Laboratory	8	24±1.1	75	8	24±1.1	75	60	14:00-15:00	1,5	AOT	Alertness<br>Cognitive performance	-	46.9
**Bauckhaus 2006**	no	yes	Laboratory	17	20.3±0.4	-	17	20.3±0.4	-	60	14:45-15:45	15	PAL<br>MTT<br>TAP<br>DSB	Memory<br>Cognitive performance<br>Alertness<br>Memory	40	-
**Backhaus 2016**	no	yes	Laboratory	2 groups :<br>22 and 20	70.4±6.1	45.2	21	73.9±4.5	14.3	45<br>or 90	12:50-13:35<br>12:50-14:20	35, and 60<br>80, and 105	VLMT<br>MAT	Memory	45.7	-
Christy 2015	no	no	Laboratory	-	-	-	-	-	-	60	-	-	SUPA<br>ROCFT<br>Maze learning task	Memory<br>Cognitive performance<br>Cognitive performance	-	50
**Debarnot 2011**	no	yes	Laboratory	2 groups :<br>11 and 10	26±4.4	-	2 groups :<br>6 and 8	26±4.4	-	20<br>or 90	14:00-14:20<br>14:00-15:30	30<br>100	Sequential finger-tapping task	Memory	31.4	-
Hayashi 1999	yes	no	Laboratory	7	20.6	43	7	20.6	43	20	14:00-14:20	40, 100, and 160	Logical reasoning<br>Addition<br>Visual detection<br>Auditory vigilance	Cognitive performance	-	37.5
Hayashi 1999	yes	no	Laboratory	10	20.7	-	10	20.7	-	20	12:20-12:40	20, 80, 140, 200, and 260	Logical reasoning<br>Addition<br>Visual detection<br>Auditory vigilance	Cognitive performance	-	50
Lahl 2008	yes	no	Laboratory	2 groups :<br>25 and 14	24.4	-	2 groups :<br>25 and 14	24.4	-	6<br>or 50	13:30-13:36<br>13:30-14:20	10<br>54	Memory task	Memory	-	37.5
**Lau 2010**	no	yes	Laboratory	14	21.2±1.1	50	17	21.21±1.1	41	90	13:00-14:30	125	Direct associative memory	Memory	40	-
Lau 2011	no	no	Laboratory	2 groups :<br>15 and 11	18.98	30.8	2 groups :<br>14 and 11	18.98	32	90	12:00-13:30	30	Cued recall task<br>Multiple-choice task<br>Radical task	Memory	-	34.4
**Milner 2006**	no	yes	Laboratory	18	20.2±2.27	-	17	20.2±2.27	-	20	14:40-15:00	30<br>50	Ball and cup test<br>Auditory oddball task	Alertness	57.1	-
**Takahashi 2004**	yes	no	Laboratory	8	49	-	8	49	-	15	12:30-12:45	30, 135 and 135	Choice reaction time<br>Alertness scale	Alertness	-	40.6
**Tucker 2006**	no	no	Laboratory	12	20.1	50	17	20.1	41	60	13:00-14:00	240	Paired associates task<br>MTT<br>DSB	Memory<br>Cognitive performance<br>Memory	-	34.4
**Tucker 2008**	no	no	Laboratory	16	23.3	-	17	23.3	-	60	13:00-14:00	125	SUPA<br>Maze learning task	Memory<br>Cognitive performance	-	37.5
**Van Schalkwijk 2017**	no	yes	Laboratory	2 groups :<br>18 and 17	23.3±2.4	52.6	2 groups :<br>22 and 19	23.3±2.4	52.6	90	14:00-15:30	65	WPT<br>MTT	Memory<br>Cognitive performance	42.9	-

AOT, Arrow orientation task; **CPT II**, Conner's Continuous Performance Test Version 5; **DSB**, Digit span backwards task; **EEG**, Electroencephalographic; **MAT**, Motor adaptation task; **MTT**, Mirror Tracing Task; **PAL**, Paired associates learning; **ROCFT**, Rey–Osterrieth complex figure test; **SUPA**, Semantically unrelated paired associates; **TAP**, Test of Attentional Performance; **VLMT**, Verbal learning and memory test; **WPT**, Word-pair task. **Studies in bold:** studies included in meta-analysis.
